# Strategies to Interfere with Tumor Metabolism through the Interplay of Innate and Adaptive Immunity

**DOI:** 10.3390/cells8050445

**Published:** 2019-05-11

**Authors:** Javier Mora, Christina Mertens, Julia K. Meier, Dominik C. Fuhrmann, Bernhard Brüne, Michaela Jung

**Affiliations:** 1 Institute of Biochemistry I, Faculty of Medicine, Goethe-University Frankfurt, Theodor-Stern-Kai 7, 60590 Frankfurt, Germany; JAVIERFRANCISCO.MORA@ucr.ac.cr (J.M.); Christina.Mertens@med.uni-heidelberg.de (C.M.); meier@biochem.uni-frankfurt.de (J.K.M.); fuhrmann@biochem.uni-frankfurt.de (D.C.F.); b.bruene@biochem.uni-frankfurt.de (B.B.); 2 German Cancer Consortium (DKTK), Partner Site Frankfurt, Germany; 3 Project Group Translational Medicine and Pharmacology TMP, Fraunhofer Institute for Molecular Biology and Applied Ecology, 60596 Frankfurt, Germany

**Keywords:** tumor-associated macrophages, T cells, hypoxia, cancer cell metabolism, iron metabolism, iron chelator

## Abstract

The inflammatory tumor microenvironment is an important regulator of carcinogenesis. Tumor-infiltrating immune cells promote each step of tumor development, exerting crucial functions from initiation, early neovascularization, to metastasis. During tumor outgrowth, tumor-associated immune cells, including myeloid cells and lymphocytes, acquire a tumor-supportive, anti-inflammatory phenotype due to their interaction with tumor cells. Microenvironmental cues such as inflammation and hypoxia are mainly responsible for creating a tumor-supportive niche. Moreover, it is becoming apparent that the availability of iron within the tumor not only affects tumor growth and survival, but also the polarization of infiltrating immune cells. The interaction of tumor cells and infiltrating immune cells is multifaceted and complex, finally leading to different activation phenotypes of infiltrating immune cells regarding their functional heterogeneity and plasticity. In recent years, it was discovered that these phenotypes are mainly implicated in defining tumor outcome. Here, we discuss the role of the metabolic activation of both tumor cells and infiltrating immune cells in order to adapt their metabolism during tumor growth. Additionally, we address the role of iron availability and the hypoxic conditioning of the tumor with regard to tumor growth and we describe the relevance of therapeutic strategies to target such metabolic characteristics.

## 1. The Delicate Interplay between the Host Immunity and the Tumor

Tumors are characterized by the development of an adequate milieu, including factors and conditions that are necessary for tumor development and progression. The tumor microenvironment comprises distinct soluble and cellular components within a unique extracellular matrix [1]. This creates regions of divergent nutrient and oxygen availability, which, in turn, affect tumor biology [2]. The complexity and differences within these distinct intratumoral regions generate individual tumor microenvironments that modify the phenotype of their cellular components [3]. In the case of immune cells, especially macrophages (MΦs) and T cells, the microenvironment dictates their polarization, which is driven not only by immune mediators, but also by the different metabolites and metabolic conditions [4,5]. 

Tumorigenesis is a complex and dynamic process involving the interaction of tumor cells with tumor-infiltrating immune cells. A major immune cell population infiltrating human and experimental tumors are MΦs, with their numbers being directly associated with clinical outcome and prognosis [6,7,8,9]. Unlike the distinct function of MΦs in maintaining normal tissue homeostasis, fighting infections and eradicating damaged or transformed cells, immune surveillance is damped within the tumor. Tumor cells shape the MΦ phenotype by secreting a variety of different factors that provoke the polarization of tumor-associated MΦs (TAMs) towards a tumor-supporting, rather anti-inflammatory and immune-suppressive phenotype. The activation phenotypes of MΦs range from a classical pro-inflammatory to the alternative anti-inflammatory status. TAMs are associated with an anti-inflammatory phenotype, showing pro-tumor activities such as the recruitment of anti-inflammatory immune cells, dampening T cell responses, as well as promoting tumor invasion and metastasis. TAM polarization is driven by cytokines such as transforming growth factor (TGF)β, interleukin (IL)-10, IL-13 and IL-4, growth factors such as epidermal growth factor (EGF), macrophage colony stimulating factor (M-CSF), and granulocyte-macrophage colony-stimulating factor (GM-CSF) [10] as well as lipid mediators such as sphingosine-1-phosphate (S1P) [11] or prostaglandin E2 (PGE_2_) [12]. 

However, not only are tumor-cell derived mediators able to skew the TAM phenotype, but also direct cell-cell interaction between MΦs and tumor cells. Hereby, dying tumor cells play a pivotal role [13]. Dying tumor cells undergoing programmed cell death either by apoptosis or necroptosis are sensed and phagocytosed by MΦs. In turn, this activates functional programs in MΦs, such as inducing matrix remodeling, neovascularization, or the inhibition of anti-tumor immunity [13,14]. These are physiological characteristics of MΦs during wound healing and regeneration [15,16] and adds to the notion that cancer might be considered as “wounds that do not heal” [17]. However, the crosstalk of MΦs and dying tumor cells not only induces functional consequences to the MΦ phenotype, but also results in a high metabolic challenge for MΦs through the recycling of the metabolic load after engulfment of cell debris that needs to be handled and tightly controlled by MΦs [18]. As such, MΦs serve as a turnover hub to acquire, recycle, and redistribute metabolic intermediates as well as metabolically relevant substances such as iron. Thus, the metabolic signature also plays a crucial role in MΦ polarization, including the level of fatty acid oxidation [19], hypoxia inducible factor (HIF)-1α activation, iron availability, or lactate exposure [20]. The combination of these signals within the complex tumor scenario makes the polarization of TAMs a dynamic process [21]. This is also related to the spatial distribution of TAMs within the tumor, with distinct TAM subpopulations being found in different regions, largely depending on oxygen and nutrient availability [22]. 

The metabolic signature of the microenvironment is also responsible for the development of the immunosuppressive nature of tumors. This is mainly associated with the polarization of T cells towards a T regulatory phenotype (Treg), which promotes T cell anergy and exhaustion as well as the production of anti-inflammatory cytokines and expression of immune checkpoints such as programmed cell death protein 1 (PD-1). Along these lines, it was recently found that T cell suppression is dependent on tryptophan depletion by the expression of indoleamine-2,3-dioxygenase (IDO) and HIF-1α, which, in turn, reduced T cell proliferation and activity [23], whereas inhibitory receptors are upregulated on activated T cells [24]. Therefore, a better understanding of the metabolic needs, but also the metabolic turnover of both tumor cells and infiltrating immune cells, is urgently needed to efficiently adapt cancer therapy.

## 2. Cancer Cell Metabolism—Nutrient Plasticity at a Glance

In the 1920s, Otto Warburg published that tumor cells rely on glycolysis as the central metabolic pathway, which was accompanied by an increased lactate production [25]. This observation, named the Warburg effect, is still referred to almost a century later. Today, we know that tumor cell metabolism is more than just a simple shift towards glycolysis [26]. Mitochondrial metabolism including the tricarboxylic acid (TCA) cycle and oxidative phosphorylation (OXPHOS) were shown to play a pivotal role in cancer progression and adaptation to low nutrient availability and low oxygen tension [27,28]. The TCA cycle represents a central part of metabolism. Glucose and acetyl-CoA enter the TCA cycle, whereas glutamine joins the cycle via glutamate and α-ketoglutarate [29]. Besides glucose and glutamine, tumor cells use unconventional nutrients such as lactate and ketone bodies to gain crucial metabolites [30,31]. Both lactate and ketone bodies are processed to acetyl-CoA and enter the TCA cycle. These are very general modifications of tumor cells concerning metabolite dependency. To obtain more detailed information, the microenvironmental niches have to be taken into account, because they require special adaptations [5]. The tumor is composed of well-vascularized regions with sufficient levels of nutrients and oxygen, but also regions where hypoxia, nutrient deprivation, or inflammatory conditions prevail [2,32]. Further, the context of the tumor in terms of tumor type, severity, and stage appears crucial for understanding the continuous metabolic adaptation processes occurring in both cancer cells as well as cells of the tumor stroma. 

### 2.1. Adaptations of Tumor Cell Metabolism under Hypoxia

**Figure 1:**
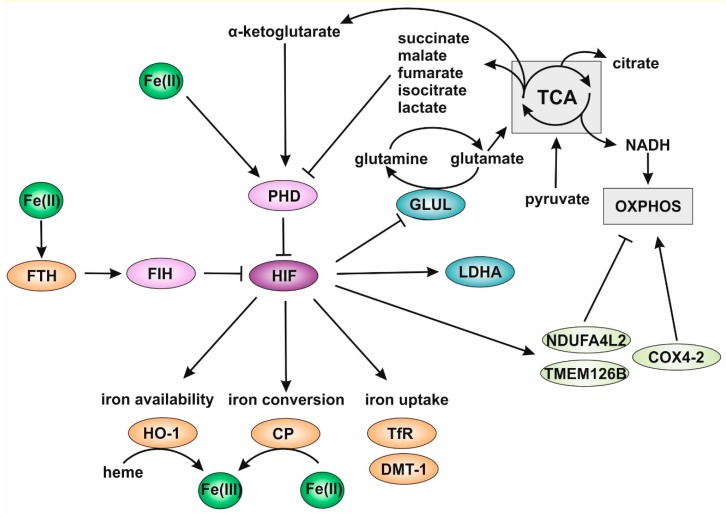
Hypoxia inducible factor (HIF) as a central mediator in iron homeostasis. Degradation of hypoxia inducible factors (HIFs) is mediated by prolyl hydroxylases (PHDs), which are regulated by various tricarboxylic acid (TCA) cycle metabolites. TCA is fueled by glutamine and pyruvate from glycolysis and provides electrons via NADH for oxidative phosphorylation (OXPHOS). OXPHOS is regulated by NADH dehydrogenase [ubiquinone] 1 alpha subcomplex subunit 4-like 2 (NDUFA4L2), the complex I assembly factor TMEM126B, and cytochrome c oxidase subunit (COX) 4-2. Inhibition of glutamine synthase (GLUL) under hypoxia could facilitate fuel for TCA by glutamate. While ferrous iron (Fe(II)) and α-ketoglutarate are essential cofactors for PHDs, succinate, malate, fumarate, isocitrate, and lactate act as inhibitors. Lactate is produced by the HIF-target lactate dehydrogenase (LDH). Another HIF limiting protein is factor inhibiting HIF (FIH), which is regulated by binding of the iron storage protein ferritin heavy chain (FTH). HIF in turn modulates iron metabolism by enhancing the transcription of heme oxygenase 1 (HO-1), ceruloplasmin (CP), the iron transporter transferrin (TfR) and divalent metal transporter 1 (DMT-1). HO-1 removes ferric iron (Fe(III)) from heme and CP converts extracellular Fe(II) to Fe(III).

Solid tumors are characterized by hypoxic regions. Hypoxia arises, when the cellular oxygen demand is higher than the supply, which can be disturbed by insufficient vascularization. Cells within a hypoxic area have to adapt their metabolism and energy production to low oxygen levels. Major conductors of the response to hypoxia are HIFs. HIFs are heterodimeric transcription factors, which consist of an α- and a corresponding β-subunit. When sufficient oxygen is available, prolylhydroxylases (PHD) hydroxylate the constitutively expressed α-subunits and mark them for degradation [33,34]. In contrast, a lack of oxygen sustains PHD function and facilitates the stabilization of HIF-α, which then translocates to the nucleus and dimerizes with the β-subunit as well as other accessory proteins. Three isoforms of the HIF-α subunit are known (HIF-1α, -2α, and -3α), of which HIF-1α and -2α are best explored and account for the majority of metabolic adaptations under hypoxia. At the metabolite level, knockdown experiments of HIF-1α and -2α as well as a knockdown of both isoforms in tumors grown from MDA-MB-231 cells indicated that the elimination of both isoforms efficiently attenuated Warburg-like metabolic adaptation and avoided compensatory effects between HIF isoforms [35]. Further, HIF is known to increase glucose uptake by glucose transporter 1 (GLUT1) and lactate production by increasing lactate dehydrogenase (LDH) A, and at the same time reducing pyruvate dehydrogenase by inducing pyruvate dehydrogenase kinase expression [36,37,38] (Figure 1). This attenuated pyruvate uptake into mitochondria. As a consequence, this reduced the major fuel source of the TCA cycle. The Warburg phenotype was abolished in hepatocellular carcinoma cells by expressing micro RNA 3662, which decreased not only the HIF-1α protein, but also the corresponding induction of GLUT1, hexokinase 2, pyruvate kinase isoenzyme M2 (PKM2), and LDHA [39]. Further, respiratory rates increased and extracellular acidification as a result of lactate secretion decreased. Interestingly, TCA cycle metabolites such as fumarate, succinate, oxaloacetate, lactate, and pyruvate are known to attenuate PHD function, and consequently, support HIF stabilization [40]. In contrast, α-ketoglutarate is a crucial substrate for PHDs, thus promoting HIF degradation [41]. This underlines the close connection between HIF-regulation and cellular metabolism. The role of HIF in adaptation to hypoxia appears crucial, since the growth of breast cancer cells was reduced, when glucose uptake was suppressed [42]. Along these lines, lactate production and secretion was enhanced, which corresponds to Warburg’s initial observation, and was abolished by the disruption of glucose-6-phosphate isomerase (GPI) in LS174T and B16 cancer cell lines [43]. Interestingly, OXPHOS was increased in GPI knockout cells and appeared to rescue the proliferation of these cells under normoxic conditions, while they stopped growing under hypoxia. Supporting these data, interference with glycolysis by the depletion of LDHA and B shifted glycolysis to OXPHOS [44]. This corroborates several reports of modifications within the respiratory chain, especially complex I under hypoxia [45,46]. For example, the HIF-1-mediated induction and incorporation of NADH dehydrogenase [ubiquinone] 1 alpha subcomplex subunit 4-like 2 (NDUFA4L2) is thought to be responsible for a deficient respiratory chain under hypoxia [47,48]. Further, under chronic hypoxia, the assembly of complex I was disturbed by proteasome-mediated degradation of the complex I assembly factor TMEM126B [49]. Besides complex I, also complex IV was shown to be altered by the HIF-dependent induction of cytochrome c oxidase subunit (COX) 4-2, which optimized the oxygen usage of the complex [50]. The impact of hypoxia and HIF on the respiratory chain was reviewed in [41].

Glutamine is also a central metabolite which is needed to ensure cell proliferation and survival. Under hypoxia, glutamine uptake is increased. Glutamine is processed to glutamate and further to α-ketoglutarate and citrate via isocitrate dehydrogenase [51]. ATP-citrate lyase (ACLY) turns citrate into acetyl-CoA under hypoxic conditions, which is then further used for lipid synthesis. The remaining oxaloacetate is turned into aspartate via glutamic-oxaloacetic transaminase 1 and afterwards by carbamoylphosphat-synthetase (CPS II), aspartat-transcarbamoylase and dihydroorotase (CAD) together with carbamoylphosphate into dihydroorotate and by dihydroorotate dehydrogenase (DHODH) to orotate [52]. This pathway is claimed to be an alternative to the urea cycle and protects cells from toxic ammonia. In breast cancer cells, glutamine synthetase (GLUL) was identified as strongly down-regulated by hypoxia and correlated to HIF stabilization [53]. In patients with severe breast tumors, HIF was highly abundant and associated with reduced GLUL levels. Interestingly, acute myeloid leukemia cells had increased GLUL mRNA levels under hypoxic conditions [54]. Taking these observations into account, it becomes obvious that due to the heterogeneity in the tumor microenvironment, it is difficult to define a specific metabolic state for tumors. Beyond distinct pathways, the distribution and use of metabolites and cofactors is crucial. 

As a cofactor for proteins and regulator of protein expression, iron is a central mediator in cellular metabolism and adaptation. In the context of hypoxia, iron is crucial for PHD function and, consequently, for degradation of the HIF-α subunits [55]. Chelation of iron by CP94 in rat kidneys and human kidney cells stabilized HIF-α by the inhibition of PHD activity and induced target genes such as erythropoietin [56]. Genetic disruption of the iron-regulatory proteins vacuolar-ATPase complex and the assembly factors TMEM199 and CCDC115 facilitated HIF stabilization, even under normoxic conditions [57]. Further, Jin and coworkers showed decreased HIF-1α stabilization by binding ferritin heavy chain (FTH) to the factor inhibiting HIF (FIH) under iron-enriched conditions [58]. In contrast, a low iron availability reduced the binding of FTH to FIH and, consequently, to an increased HIF stabilization. These studies illustrate a pivotal role of iron in regulating hypoxic responses. A summarizing scheme of hypoxic adaptation and the role of hypoxia for cellular iron metabolism is provided in Figure 1.

### 2.2. Impact of Iron on OXPHOS and TCA

Besides its role in a pathophysiological- or hypoxia-related context, iron plays a pivotal role in the function of OXPHOS and TCA. Iron is incorporated in subunits of complex I, II, III and IV of the respiratory chain, ensures the transport of electrons, and thus the maintenance of the mitochondrial membrane potential and ATP production. On the other hand, a dietary iron exposure caused a significant impairment in OXPHOS capacity and was paralleled by an iron-mediated induction of oxidative stress in mitochondria [59]. A decreased abundance of respiratory chain complexes was observed in primary human cardiac myocytes, which were exposed to reduced iron concentrations. This was compensated by an increased expression of glycolytic enzymes and led to an elevated lactate production by LDHA. Interestingly, iron-enriched conditions decreased the respiratory chain complexes as well, but failed to increase LDHA expression and consequently lactate production [60]. The activity of TCA enzymes is strongly dependent on iron. Thus, the activity of the mitochondrial aconitase is reduced through addition of the iron chelator desferrioxamine (DFO). Iron also positively affects the TCA enzymes, citrate synthase, isocitrate dehydrogenase, and succinate dehydrogenase, while DFO decreased their activity. Consequently, iron supplementation resulted in the increased formation of reducing equivalents (NADH) by TCA, which are used as electron donors by OXPHOS. An increased ATP production by OXPHOS led to a downregulation of glucose utilization. Upon DFO-mediated iron depletion, glycolysis and lactate formation were significantly increased in order to compensate for the impaired OXPHOS [61,62]. 

### 2.3. Tumor Cell Iron Metabolism

Besides its role in HIF-regulation, iron is an essential trace element in all living organisms. Iron overload and iron depletion can severely affect physiological processes such as erythropoiesis or development. The liver represents the major iron storage organ and is most susceptible to injuries due to iron overload [63]. For this reason, iron homeostasis must be tightly regulated, and free iron only occurs transiently in the serum. After the absorption of iron from the intestine into the blood, iron immediately binds transferrin (Tf), which is then transported via the plasma to the tissues. Uptake of Tf-bound iron is ensured by binding the transferrin receptor (TfR), which was also found to be up-regulated in tumor cells. In response to hypoxia, the expression of Tf is increased due to the enhanced transport of iron into erythroid tissues, where it is used for heme synthesis [64]. Intracellular iron is rapidly stored in ferritin to overcome ROS generation, which is induced by excess free intracellular iron. FTH and FT light (FTL) build up the ferritin complex, able to store up to 4500 iron atoms in a ferrihydrite mineral core [65]. In the tumor microenvironment, the upregulation of FT in stromal cells has been described to contribute to tumor growth, most likely by providing iron to tumor cells [31]. Compared to healthy cells, cancer cells not only accumulate iron by down-regulating iron release mechanisms through degradation of the iron exporter FPN, but also show a higher iron turnover and mobilization, which is associated with their enhanced metabolic needs. These adaptations are correlated with the development of more aggressive tumors and poor patient survival [66,67]. Thus, the malignant state is often associated with a deregulated cellular and systemic iron metabolism. Increasing iron uptake into tumor cells via the TfR provides more iron for the enhanced metabolic and proliferative purposes of cancer cells [68]. Cellular iron homeostasis is controlled by RNA-binding proteins and RNA-binding elements, constituting a post-transcriptional gene expression regulation system called iron regulatory protein (IRP)/iron-responsive element (IRE) system [69]. IRP1 and 2 as well as the cis-regulatory RNA elements, IREs, present in mRNAs encoding for proteins involved in iron homeostasis, coordinate iron uptake, utilization, and storage dependent on intracellular iron levels. IRP/IRE interactions regulate the expression of proteins involved in iron acquisition (divalent metal transporter-1 (DMT-1), TfR), iron storage (FTH, FTL), and iron utilization (erythroid 5-aminolevulinic acid synthase (Alas2)), as well as energy supply (mitochondrial aconitase (Aco2)) and iron export (FPN) [69]. 

Besides cellular iron homeostasis, systemic iron homeostasis is regulated by the hepcidin/FPN system [70]. Hepcidin is a liver-specific, iron-sensing protein and considered the master regulator of iron homeostasis. It is secreted in response to inflammation or under conditions of iron excess. To limit iron export, hepcidin binds to the iron exporter, FPN, whereby its internalization and degradation is induced and the delivery of iron from the digestive tract to the blood is blocked [71]. Similarly, hepcidin binds to FPN expressed on reticuloendothelial macrophages located in the spleen and bone marrow, where it blocks iron recycling, which further limits iron availability [66]. Increased hepcidin levels cause anemia, while decreased expression is a causative feature in most primary iron overload diseases [72,73]. In cancer cells, the local expression of hepcidin accounts for intracellular iron accumulation by binding to FPN. This results in an increase of metabolically available iron within tumor cells through decreased iron efflux [70,71]. Under physiological conditions, hepcidin is induced in hepatocytes via a bone morphogenetic protein (BMP)-mediated pathway, when intracellular iron storage and circulating levels of iron are high, and is secreted into the circulation [74]. Hepcidin upregulation during tumor growth is induced in response to inflammatory stimuli such as IL-6, which, in turn, often leads to anemia of chronic disease [75]. Besides the altered iron metabolism and enhanced iron sequestration due to an increase in hepcidin expression, in cancer patients and granulomatous patients (CGD) that suffer from primary immunodeficiency, the release of inflammatory cytokines also reduces red blood cell survival, thereby reinforcing the progression of anemia. Anemia and hypoxia are both associated with a dramatic decrease in liver hepcidin expression, which may account for the increased demand of iron [76]. After correction of anemia, rectification of the iron status promotes HIF degradation. However, if iron remains deficient due to persistent anemia, HIF stabilization persists and HIF target gene expression is maintained to ensure increased intestinal iron absorption to replete iron stores. 

Iron homeostasis is regulated by many factors and the IRP/IRE system is not the only way to regulate iron homeostasis, which is divided into multilayered regulation. HIF has also been reported to regulate intracellular iron by binding to HIF-responsive elements (HRE) located within iron-related genes, such as TfR and hemeoxygenase-1 (HO-1). Initially, the hypoxia-mediated upregulation of Tf/TfR was thought to arise solely from IRE/IRP activity, but Lok et al. and Tacchini et al. independently showed that the TfR is flanked by a HRE, and that it is a hypoxia-inducible HIF-1α target gene [77,78]. The ferroxidase ceruloplasmin (CP), which is required to oxidize ferrous (Fe^2+^) to ferric (Fe^3+^) iron, was also shown to be a HIF-1α target gene apart from its regulation through the IRP/IRE system and is required for efficient binding of iron to Tf [79]. The HIF-1α-dependent induction of CP, triggered by iron deficiency, supports iron supply to erythroid cells via loading of iron onto Tf. Additionally, hypoxia is one of the strongest signals for angiogenesis in tumors, initiating the formation of a vascular network for nutrient and oxygen delivery to the tumor site [80,81]. The so called “angiogenic switch” is induced by HIF-1α through activating the transcription of vascular endothelial growth factor (VEGF)A, angiopoietin 2, cyclooxygenase 2, stromal-derived factor 1, and stem cell factor [82,83,84]. Along these lines, activated HIF-2α has been shown to promote colorectal cancer by inducing DMT-1 expression, thereby increasing iron uptake, which, in turn, contributed to enhanced iron accumulation in tumors [85]. Nevertheless, the majority of iron is reutilized from cellular hemoproteins. In response to hypoxia, HIF-1α has been reported to mediate the transcriptional activation of HO-1 [86]. HO-1 plays an important role by processing heme to free iron, biliverdin, and carbon monoxide. This pivotal role is highlighted in HO-1 knockout mice that develop severe anemia associated with low serum iron levels [87]. Also, hepcidin repression during deficiency-induced anemia is associated with an induction of HIF-1α stabilization, further triggering anemia of chronic disease [88], which is also seen in cancer patients. In a number of studies, the expression of different iron-regulated genes such as TfR [76], FTL [77,78], and IRP2 [79] in tumor cells was correlated with a poor prognosis and a higher tumor grade, leading to increased chemoresistance in cancer patients. 

Targeting iron metabolism, either in cancer cells or in tumor-infiltrating immune cells, might represent one major therapeutic improvement in combination with conventional therapy. 

## 3. Anti-Tumor Therapies—Immunotherapeutic Approaches and Beyond 

### 3.1. Targeting Iron Metabolism in Cancer—A Tug of War 

Iron handling in the tumor microenvironment emerges as an important aspect of tumorigenesis. It is not surprising to find increased iron levels in cancer cells, since iron is also responsible for DNA synthesis and cell cycle progression [89]. Due to their enhanced metabolism, neoplastic cells have a higher need for iron and it has been shown that perturbation of cellular iron uptake proteins arrests cell growth [6]. Additionally, a number of studies have shown that iron is able to drive tumorigenesis. Patients regularly donating blood have a decrease in total body iron and a reduced risk of cancer, while patients with iron-overload disease are at an increased risk [90]. Excess iron occurs during several diseases, including diabetes, neurodegenerative disorders, and heart disease [91]. The body does not have a system for effective iron excretion, whereby excess systemic iron fuels the development of iron [6] storage diseases such as hereditary hemochromatosis or iron-overload diseases such as β-thalassemia and Friedrich’s ataxia. In particular, iron deposits are found in the liver, pancreas and heart. Iron overload-mediated ROS generation causes, in turn irreversible tissue damage and fibrosis [92]. To confine iron during iron-overload conditions, natural and synthetic iron chelators, such as desferrioxamine (DFO), deferiprone, and deferasirox have been utilized for decades; oral iron chelators are rather recent developments for the clinical treatment of iron overload due to chronic blood transfusion therapy [93]. More recently, iron chelators have been subjected to preclinical and clinical trials in order to evaluate their anti-neoplastic potential. DFO for example inhibits proliferation and induces apoptosis by arresting the cell cycle [6]. Nevertheless, a detailed knowledge of the effects of chelators within the tumor microenvironment is still lacking. Current clinical chelators primarily bind labile plasma iron and are not designed to specifically target iron in either tumor cells or MΦs. In combination with a high hydrophilicity and unfavorable pharmacokinetics, it is nearly impossible to reach an effective intratumoral concentration without unacceptable, severe side effects [94]. Therefore, a critical step towards successful iron-chelation therapy for cancer treatment is targeting iron specifically in the tumor as compared to healthy tissue or the extracellular space. Prodrug approaches as well as targeted delivery methods are expected to enhance selectivity and circumvent systemic toxicity as well as severe side effects, often occurring during clinical trials of novel iron chelation therapies. Along these lines, Akam et al. developed a prochelation approach using a disulfide switch for the intracellular activation of a thiosemicarbazone iron chelator [95,96,97]. The higher levels of reduced glutathione in cancer cells, especially in hypoxic tumor areas, causes the reduction of the prochelator in the intracellular milieu and the generation of a thiolate, resulting in the formation of a low-spin Fe(III) complex [95]. By taking advantage of the Warburg effect in tumor cells, the increased uptake of glucose and the higher glycolytic rates in cancer cells compared to non-malignant cells, glucose-conjugated prochelators might be used to deliver the thiosemicarbazone iron chelator directly to cancer cells [96,98]. However, it still remains to be investigated whether these classes of iron chelators are also effective in the complex in vivo situation of experimental tumors and whether their targeting potential is maintained within a microenvironment of variable composition of a variety of immune and stromal cells.

### 3.2. Tumor-Associated Macrophages—Promising Targets or Major Players in Tumor Immunotherapy?

**Figure 2:**
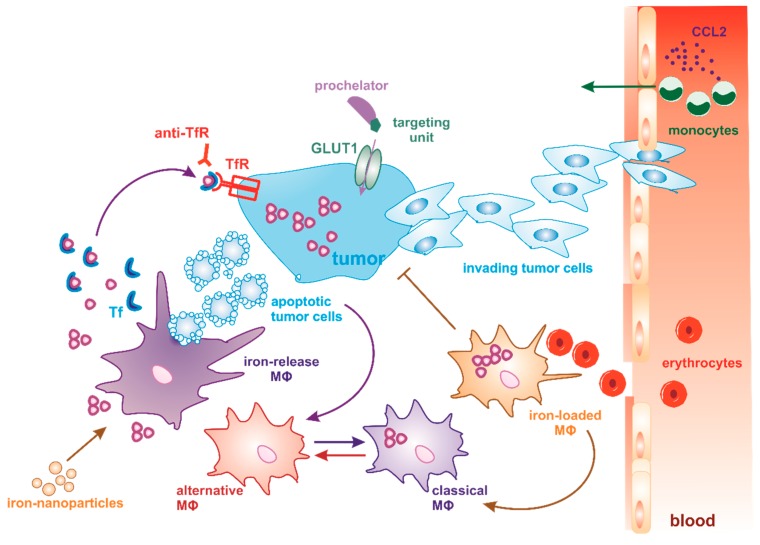
Dynamics and targeting of MΦ phenotypes in the tumor microenvironment. Macrophages (MΦs) can be targeted to reprogram the immunosuppressive tumor microenvironment and consequently enhance anti-tumor response. One strategy is to prevent the systemic mobilization and recruitment of monocytes to the tumor site by specifically blocking chemokine gradients such as CCL2. Instead of completely depleting MΦs, therapies aim at reprogramming alternatively polarized MΦs towards the anti-tumor, classical MΦ phenotype. With regard to their iron-regulated gene profile, two different MΦ phenotypes are distinguished. The iron-release phenotype is determined by the phagocytosis of apoptotic tumor cells, whereby iron is recycled from engulfed cells and secreted to the microenvironment, where it is rapidly bound to transferrin (Tf). Tf-bound iron is then taken up by tumor cells through the Tf receptor (TfR) and promotes tumor proliferation and growth. The engulfment of apoptotic tumor cells also promotes the anti-inflammatory MΦ phenotype. In order to control iron availability in tumor cells, targeted iron-chelation therapy (prochelator strategy) is used. This is accomplished by conjugating the prochelator with a glucose targeting unit, which is recognized by glucose transporter (GLUT1) on tumor cells. Another possibility is the use of TfR antibodies, which has shown strong anti-neoplastic effects. In contrast, iron-loaded MΦs reside in close vicinity to tumor vessels, where they are exposed to high levels of erythrocytes leaking into the tumor. Iron-loaded MΦs also adopt a pro-inflammatory phenotype. Iron nanoparticles are currently under investigation to reduce pro-tumor MΦ functions and, thus, tumor growth.

TAMs acquire a tumor promoting phenotype through their interaction with tumor cells and signals delivered by the tumor microenvironment. Different strategies have been proposed to target the pro-tumor function of TAMs [10], including the inhibition of recruitment, depletion, and reprogramming towards a tumoricidal phenotype. This includes the inhibition of monocyte recruitment into the tumor or the complete depletion of TAM. For TAM depletion, chemical compounds such as bisphosphonates, including clodronate, are often used. After phagocytosis of these liposomes, clodronate is metabolized and is cytotoxic for MΦs [99]. In patients as well as in mouse models, the depletion of TAM resulted in tumor regression. Nevertheless, the systemic depletion of macrophages renders the organism more sensitive for infections. A future perspective could be to target TAM, without affecting other MΦ subtypes throughout the body. The inhibition of CCL2/C–C chemokine receptor type 2 by monoclonal antibodies, for example carlumab, or by interfering with its synthesis by trabectedin, blocks the recruitment of monocytes from the bone marrow or the blood to the tumor site (Figure 2). Some of these treatments that showed high efficiency in experimental and pre-clinical animal models are currently in clinical trials for the treatment of different types of cancer [100,101,102]. Instead of blocking MΦ attraction to the tumor site or the depletion of MΦs, other approaches followed the idea of shifting the pro-tumor, anti-inflammatory TAM phenotype towards an anti-tumor, pro-inflammatory MΦ phenotype [103,104,105]. The major transcription factor involved in TAM reprogramming is STAT3 [106]. Anti-cancer therapy using multikinase inhibitors, e.g., sorafenib and sunitinib, were not only effective in reducing tumor growth, but also inhibited STAT3 expression in MΦs, whereby IL-10 secretion was blocked and IL-12 was induced, thus suggesting an immune activating TAM phenotype [107] Moreover, treatment of glioblastoma patients with WP1066 inhibited STAT3 activation and induced the expression of pro-inflammatory cytokines in MΦs, whereby T cells were activated, finally reducing immunetolerance [108]. Even though promising results have been observed using the inhibition of recruitment, depletion and reprogramming of TAM, it is important to consider that the immunological profile of MΦs is not only controlled by transcription factors and a specialized gene profile but it is also influenced by their metabolic state. Factors such as oxygen tension, metabolite accumulation, active metabolic pathways, and local iron metabolism modulate the phenotypic polarization of MΦs and their role in tumor biology. 

TAM polarization at later stages of tumor development is determined by metabolites such as lactate, which potentiate their anti-inflammatory properties. Lactate has the ability to induce angiogenesis as well as cell migration, since lactate produced by cancer cells induces the expression of VEGFA in a HIF-1α-dependent manner [20]. Additionally, lactate is found in the hypoxic regions of the tumor and its accumulation correlated with poor prognosis. In tumors, lactate production is enhanced, even in the presence of oxygen due to metabolic alterations [109], making it a suitable target to modify the TAM activation profile. Besides accumulation of metabolites in the microenvironment, the metabolic pathways activated within MΦs modulate their activation phenotype. Pro-inflammatory MΦs are characterized by increased glycolysis, whereas anti-inflammatory MΦs switch to oxidative phosphorylation and fatty acid oxidation [110]. This metabolic switch is controlled by factors such as mTOR and HIF-1α. Thus, modifications in these pathways could reverse the macrophage metabolism to an anti-tumor phenotype, activating pro-inflammatory pathways and inhibiting metastasis as well as angiogenesis [111]. Increased fatty acid oxidation and oxidative phosphorylation, which are determinants for pro-tumor polarization in TAM, are induced by the PI3K–AKT–mTOR pathway [112]. This pathway is also associated with promoting L-arginine metabolism, which is a key aspect of altered metabolism that promotes immunosuppression mediated by TAMs. Therefore, clinical trials targeting PI3Kγ with the agent IPI-549 are being developed to polarize TAMs towards an anti-tumoral phenotype [113].

More approaches were applied to modify the TAM phenotype aimed at modulating their iron metabolism. We recently described that pro-inflammatory MΦs show an iron-sequestration phenotype, whereas anti-inflammatory MΦs and TAMs are characterized by an iron-export phenotype [114,115]. These phenotypes have been characterized in MΦs that were exposed to apoptotic tumor cells, whereby an anti-inflammatory phenotype is induced. In contrast, in a murine lung carcinoma model, it was shown that the TAM subpopulation that resides in hemorrhagic regions is highly iron-loaded, upregulates NOS2, and is associated with a pro-inflammatory response that is toxic against malignant cells [116]. In the tumor microenvironment, such properties were speculated to be induced by heme, which is recycled in MΦs through the ingestion of red blood cells derived from leaky tumor vessels. Interestingly, the phenotype is also induced by the treatment of tumor-bearing mice with iron nanoparticles, leading to tumor suppression [116]. The relationship between these different MΦ polarization programs both in terms of iron metabolism and the MΦ activation status were previously reviewed in [117,118]. These rather opposing observations regarding the TAM iron phenotype have to be further addressed in future studies, especially taking into account the huge diversity of MΦ activation states found within the tumor microenvironment (Figure 2). Moreover, there is still very little known about the role of these iron-regulated MΦ subsets with regard to their ability of immune modulation or potential to enhance or even reverse immunosuppression and/or immune tolerance. 

Considering that TAMs are one of the major infiltrates of experimental and human tumors, it is believed that they actively contribute to the escape from immune surveillance, whereby TAMs suppress effector functions of cytotoxic T cells and, thus, also compromise novel immune checkpoint therapy.

### 3.3. T Cells at the Center Stage of Effective Anti-Tumor Immunity

**Figure 3:**
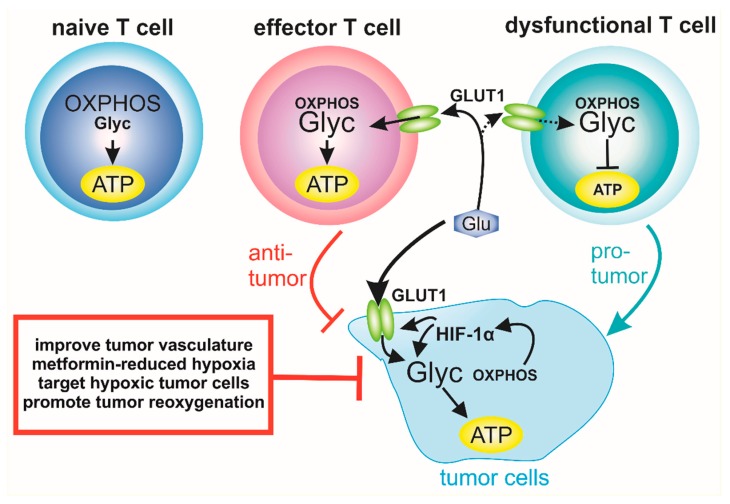
T cell Tumor cell metabolism dictates T cell effector function. Naïve T cells produce ATP predominantly by oxidative phosphorylation. After activation, a metabolic switch towards glycolysis is associated with T cell effector functions. Increased expression of the glucose transporter GLUT1 is essential for ATP production in effector T cells. Due to HIF-1α stabilization in tumor cells, GLUT1 expression is enhanced, which, in turn, leads to accumulation of glucose in tumor cells used for glycolysis, whereby OXPHOS is reduced. As a consequence, sufficient glucose is not available to maintain T cell effector functions and T cells adopt a dysfunctional phenotype due to reduced ATP production. Thus, targeting tumor hypoxia, e.g. by metformin, is a novel strategy to improve T cell function and response to immune-check point blockade.

In recent years, immune checkpoint inhibition has been explored as a promising option to treat different types of cancer. As was mentioned above, T cell exhaustion is a hallmark feature of most immune cell-infiltrated tumors. Therefore, strategies to reverse this T cell phenotype towards an active and functional status has been proven to be successful to attack and eliminate tumor cells (Figure 3). Interfering with PD-1/PD-L1 interaction has been approved for the treatment of several cancer types, nevertheless a positive response using these therapies is associated only with patients bearing tumors with certain characteristics such as high PD-L1 expression and high mutation rate. Therefore, new approaches to increase the efficacy of these therapies are necessary.

Immune-associated events such as deficient T cell infiltration, impaired antigen cross-presentation, and the recruitment of suppressive immune cell types are being explored as potential biomarkers or targets to predict and improve the response to immune checkpoint blockade [119]. Besides these options, it is important to consider T cell metabolism to improve immune activation and tumor cell elimination. Oxidative phosphorylation is the main metabolic pathway for ATP production in naive T cells, which is also maintained at a certain level upon T cell activation [110,120]. Additionally, effector T cells show a Warburg-like metabolism, activating glycolysis for ATP production even in the presence of oxygen, which is HIF-1α dependent [36,121]. HIF-1α induces the upregulation of glucose transporter GLUT1, providing the T cells enough energy to perform their effector functions [122]. Therefore, it has been proposed that the reprograming of T cell metabolism might improve their effector functions. Nevertheless, within the tumor microenvironment, alterations in both oxidative and glycolytic metabolic pathways in tumor cells interfere with T cell activation and effector functions. On the one hand, tumor-infiltrating T cells are characterized by defects in oxidative metabolism, mitochondrial biogenesis and fusion, and it has been demonstrated that metabolic reprogramming of these cells improves response to PD-1 blockade [123]. On the other hand, tumor hypoxia and HIF-1α activation in tumor cells promote glycolysis, increasing glucose uptake by proliferating tumor cells. Thus, limiting glucose availability for T cells as an energy source for their effector functions might dampen antitumor immune response [120]. Moreover, hypoxia induces the recruitment of Tregs to the tumor microenvironment through CCL28 expression on tumor cells [124,125] and the expression of Foxp3 [126], thereby promoting intratumoral immune suppression. Whereas the limitation of glucose availability compromises effector functions and the proliferation of cytotoxic T cells, Treg transcription factor Foxp3 reprograms T cell metabolism, whereby oxidative phosphorylation is enhanced in order to provide Tregs with a metabolic advantage in low glucose, but lactate-rich environments [127]. Under low glucose conditions, the glycolytic enzyme glyceraldehyde-3-phosphate dehydrogenase (GAPDH) prevents the translation of IFN-γ, a key effector molecule in tumor-infiltrating cytotoxic T cells [128,129]. Additionally, tumor hypoxia inhibits T cell proliferation and function [130] and can induce a transcriptional profile associated with resistance to checkpoint blockade [131]. Moreover, the efficacy of the PD-1 blockade is potentiated by the metformin-induced reduction of tumor hypoxia [132]. Therefore, even though T cell intracellular hypoxic signaling could be modulated to reprogram the metabolic and functional state of T cells, tumor cell hypoxia modulates the metabolic microenvironment, dampening tumor-infiltrating T cell activation and responsiveness to immune checkpoint blockade. 

In a melanoma model, T cells infiltrating tumors with both oxidative and glycolytic metabolism, or with reduced glycolytic metabolism, showed a high degree of T cell dysfunction. However, the loss of oxidative metabolism in tumor cells increased T cell activation and responsiveness to PD-1 blockade [133]. Reduced hypoxia-adapted metabolism induces mitochondrial complex I blockade, which, in turn, limits oxidative metabolism and improves the efficacy of anti-PD-1 therapy [132]. Based on the metabolic changes described, different strategies for oxygen tension normalization in the tumor microenvironment such as improving tumor vasculature [134], targeting hypoxic tumor cells [135], promoting tumor reoxygenation [136], and the metformin-dependent reduction of hypoxia [132] could be useful to complement and improve the response to immunotherapy (Figure 3). 

Summarizing, the immune checkpoint blockade represents a promising therapeutic option to use the host immune system to eliminate tumor cells. Nevertheless, the current low response rates could be improved by exploring metabolic pathways directly in T cells or in tumor cells either to use the metabolic profile as a novel biomarker to determine the likelihood of success of the immune checkpoint blockade or to target these pathways in order to create an adequate context to promote anti-tumor immune responses. 

Another aspect that compromises effective anti-tumor immunity is the development of myeloid-derived suppressor cells (MDSCs) through the exposure of myeloid cells to tumor-driving factors [137]. MDSCs are central to the creation of an immunosuppressive tumor microenvironment. They represent a heterogeneous population of immature myeloid cells with a strong immunosuppressive potential. Specifically, granulocyte-macrophage colony-stimulating factor (GM-CSF), granulocyte CSF, macrophage CSF, stem cell factor, transforming growth factor (TGF)-β, tumor necrosis factor (TNF)-α, vascular endothelial growth factor (VEGF), prostaglandin E2, cyclooxygenase 2, S100A9, S100A8, interleukin (IL)-1β, IL-6, and IL-10 are considered to be crucial for MDSC expansion [138,139,140,141,142,143]. Whereas the natural function of myeloid cells is the initiation of protective immune responses within the tumor tissue, the development of MDSCs leads to the inhibition of an effective anti-tumor reactivity of T cells and NK cells. This, in turn, immunizes tumors against chemotherapy and radiation therapy [144]. In fact, MDSCs also adapt their metabolic activation status to tumor microenvironmental needs. They upregulate fatty acid oxidation, which, in turn, supports their immunosuppressive outcome. Upregulation of fatty acid oxidation was visible by the increased key enzymes of this pathway, increased oxygen consumption, and overall increased mitochondrial mass. Interestingly, the modulation of fatty acid oxidation through pharmacological inhibition decreased the production of immunosuppressive cytokines and delayed tumor growth in a T cell-dependent manner [145]. 

Two major subpopulations of MDSCs are known and defined by their phenotypical and morphological characteristics. Monocytic MDSCs (M-MDSCs) and polymorphonuclear MDSCs (PMN-MDSCs) [139]. Both are found under pathophysiologic conditions in the bone marrow, spleen, lung, peripheral blood, and tumor tissue. PMN-MDSCs represent more than 80% of all MDSCs and are found highly expressed in tumors [135]. Among their pro-tumor functions are the induction of angiogenesis, the establishment of pre-metastatic niches, and the recruitment of other immunosuppressive cells such as Tregs [143]. MDSCs not only suppress the proliferation and cytokine secretion of T cells but promote their death by apoptosis induction. Moreover, Gr1+CD11b+ MDSCs are able to expose antigenic epitopes that induce antigen-specific T cell anergy, which is associated with CD8+ T cell tolerance within tumor tissues [146]. It was speculated that STAT3-dependent effector molecules such as IDO are induced in tumor-associated MDSCs. These observations were underlined by the fact that upregulated IDO expression and STAT3 phosphorylation in MDSCs isolated in breast cancer tissue were strongly correlated with increased Treg infiltration. Along these lines, it was shown that the inhibition of IDO or STAT3 phosphorylation are possible targets to effectively block MDSC immunosuppressive function [147].

Moreover, it was described that hypoxia, specifically through the activation of HIF-1α, is crucial for MDSC differentiation and function through the upregulation of PD-L1 on MDSCs [148]. Thus, PD-L1 inhibition as well as blocking HIF-1α might represent a promising approach to enhance cancer immunotherapy. 

## Abbreviations

CGD: chronic granulomatous disease; CP: ceruloplasmin; DFO: deferoxamine; DMT-1: divalent metal transporter-1; FPN: ferroportin; FTH: ferritin heavy chain; FTL: ferritin light chain; GLUT 1: glucose transporter 1; HIF: hypoxia inducible factor; HO-1: heme oxygenase 1; IRE: iron responsive elements; IRP: iron regulatory proteins; Lcn-2: lipocalin-2; LDH: lactate dehydrogenase; MΦ: macrophage; MDSC: myeloid-derived suppressor cell; OXPHOS: oxidative phosphorylation; PD-1: programmed cell death protein 1; PHD: prolyl hydroxylase; TAM: tumor-associated macrophage; TCA: tricarboxylic acid; Tf: transferrin; TfR: transferrin receptor; Treg: T regulatory cell.
